# Succession of microbial populations and nitrogen-fixation associated with the biodegradation of sediment-oil-agglomerates buried in a Florida sandy beach

**DOI:** 10.1038/s41598-019-55625-6

**Published:** 2019-12-18

**Authors:** Boryoung Shin, Ioana Bociu, Max Kolton, Markus Huettel, Joel E. Kostka

**Affiliations:** 1 School of Earth and Atmospheric Sciences, Georgia Institute of Technology, Atlanta, GA USA; 2 Department of Earth, Ocean and Atmospheric Science, Florida State University, Tallahassee, FL USA; 3 School of Biological Sciences, Georgia Institute of Technology, Atlanta, GA USA

**Keywords:** Marine microbiology, Soil microbiology

## Abstract

The Deepwater Horizon (DWH) oil spill contaminated coastlines from Louisiana to Florida, burying oil up to 70 cm depth in sandy beaches, posing a potential threat to environmental and human health. The dry and nutrient-poor beach sand presents a taxing environment for microbial growth, raising the question how the biodegradation of the buried oil would proceed. Here we report the results of an in-situ experiment that (i) characterized the dominant microbial communities contained in sediment oil agglomerates (SOAs) of DWH oil buried in a North Florida sandy beach, (ii) elucidated the long-term succession of the microbial populations that developed in the SOAs, and (iii) revealed the coupling of SOA degradation to nitrogen fixation. Orders of magnitude higher bacterial abundances in SOAs compared to surrounding sands distinguished SOAs as hotspots of microbial growth. Blooms of bacterial taxa with a demonstrated potential for hydrocarbon degradation (*Gammaproteobacteria, Alphaproteobacteria, Actinobacteria*) developed in the SOAs, initiating a succession of microbial populations that mirrored the evolution of the petroleum hydrocarbons. Growth of nitrogen-fixing prokaryotes or diazotrophs (*Rhizobiales* and *Frankiales*), reflected in increased abundances of nitrogenase genes (*nifH*), catalyzed biodegradation of the nitrogen-poor petroleum hydrocarbons, emphasizing nitrogen fixation as a central mechanism facilitating the recovery of sandy beaches after oil contamination.

## Introduction

In April 2010, the Deepwater Horizon oil rig exploded and sank, which led to a discharge of approximately 4.9 million barrels of crude oil into the Gulf of Mexico (GoM) at a depth of 1544 m over the course of 86 days^1,2^. Released MC252 BP oil that reached the ocean surface was transported to coastal environments, impacting approximately 965 km of beaches from east Texas to west Florida^3–7^. When weathered oil reaches the shoreline, it is generally in the form of a highly viscous, buoyant emulsion (also known as mousse), and a large portion then mixes with solids to form oil-sediment residues that have been termed tar balls, sand mats or patties, and surface residual balls^8–11^. In order to avoid confusion, we will henceforth refer to these macroscopic oil-sediment residues as sediment oil agglomerates (SOAs), according to a recent review of nomenclature^12^. SOAs are oval-shaped residues, ranging from a few millimeters to centimeters in diameter, predominantly composed of sand (75–96% by mass) with a moisture content of less than 0.5%^13–16^.

After the DWH oil spill, oil-sediment residues were trapped and buried up to meters in depth in beaches from Louisiana to Florida^13,16,17^. SOAs in highly contaminated beaches of Louisiana were found to contain elevated concentrations of recalcitrant and toxic polycyclic aromatic hydrocarbons (PAHs) including C1- and C2-phenanthrenes, C2- and C3- dibenzothiophenes, along with other high molecular weight oil components^16^. After monitoring SOAs for four years in Alabama’s beaches, Yin *et al*. (2015) showed that high molecular weight PAHs-such as chrysene and alkylated chrysenes persisted with time. Studies have also demonstrated the toxicity of SOAs due to persistence of PAHs, oxygenated hydrocarbons, environmentally persistent free radicals (EPFRs), and human pathogens such as *Vibrio vulnificus*^10,18–21^. Although British Petroleum (BP) conducted Operation Deep Clean (ODC) to mechanically remove larger SOAs from the beach surface, SOAs remained buried to 50 cm depth^4,17^. Moreover, once SOAs are buried in the sediments, degradation of SOAs cannot occur by photooxidation, a critical weathering process for hydrocarbons in surficial environments^22,23^. Therefore, even after cleanup efforts, SOAs containing toxic PAHs can persist in the beach system for years and represent a potential long-term risk to ecosystem and human health^14^.

The biodegradation of larger, macroscopic oil-sediment residues such as SOAs is likely to be distinct from that of smaller oil droplets or particles in coastal zones^13,16,24^. The smaller surface area to volume ratio of SOAs may limit the access of hydrocarbons for biodegradation^25^. Depending on the porosity of the aggregate, biodegradation is also likely to be limited by the delivery of substrates, oxygen and nutrients, to sites where microorganisms mediate enzymatic breakdown of hydrocarbons. For example, Elango *et al*. (2014) observed that the C/N molar ratio, often used as a diagnostic variable for hydrocarbon biodegradability, ranged from 111 to 474 in SOAs, which is well above optimal C/N ratios (approximately 60) for aerobic hydrocarbon degradation^26^. These findings suggest that bioavailable nutrients are often a limiting factor for microbial SOA degradation. Pure culture studies have shown that some hydrocarbon-degrading bacteria have the potential to fix nitrogen^27–30^. However, a direct linkage between SOA degradation and nitrogen fixation is lacking.

Although the impacts of oil contamination on marine microbial communities are well documented, few studies have addressed the microbial community dynamics associated with SOAs that are often trapped in coastal ecosystems. Especially in the supratidal zone of beach sand environments, low moisture content, nutrient availability, and a low surface to volume ratio of larger residues may limit bacterial hydrocarbon degradation^31^. Thus, the main objectives of this study were to (i) identify the dominant microbial organisms that colonize SOAs buried in dry northeastern Gulf of Mexico beach sand (ii) elucidate long-term succession of microbial communities that recruit onto these SOAs, and (iii) to explore the potential coupling of SOA degradation and nitrogen fixation buried in dry beach sand. This study used SOAs that were collected at Pensacola Beach after the DWH oil spill. Standardized aliquots of this material were buried in Pensacola Beach sand and monitored for the succession of microbial communities, and nitrogen fixation potential for over 3 years.

## Results and Discussion

**Figure 1:**
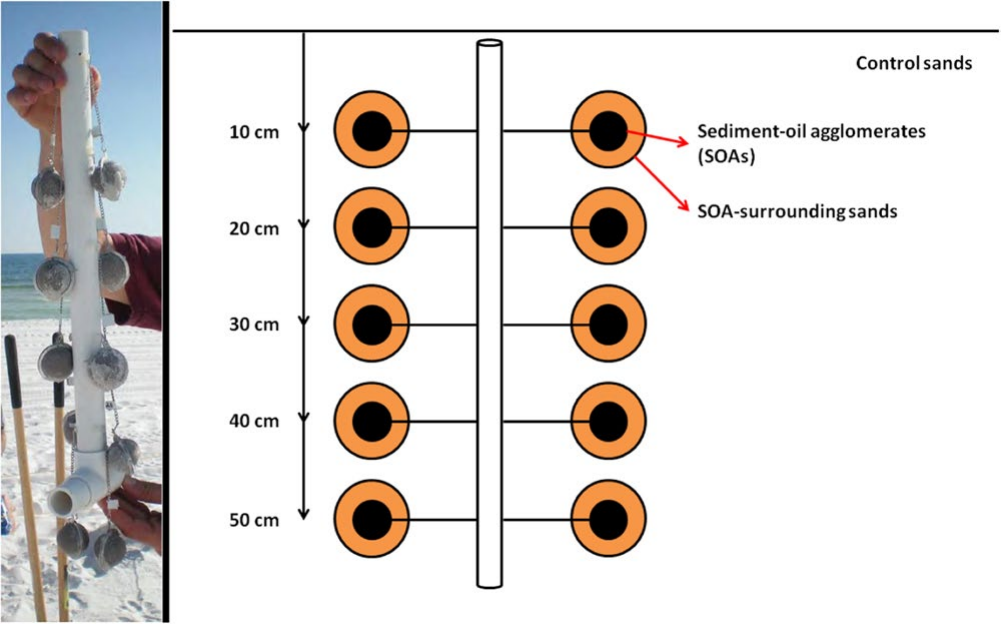
Experimental design employed to investigate the *in situ* biodegradation of sediment oil agglomerates (SOAs), showing replicate SOAs attached to PVC pipe at Pensacola Beach.

In this study, an *in situ* experiment was conducted whereby standardized sediment-oil-agglomerates (sSOA) in stainless steel meshballs (3.8 cm diameter) were attached to PVC pipe and buried in the Pensacola Beach supratidal zone from 10 cm to 50 cm sediment depth (Fig. 1). Ten of these arrays were buried on 22 October, 2010 and retrieved 41, 89, 131, 181, 235, 279, 327, 445, 735, and 1152 days after burial. SOAs along with sandy sediment surrounding each of the sSOAs were collected to characterize the impacts to indigenous microbial communities. Control sands that showed no oil contamination were also collected from the surface of a nearby sand dune after 41, 89, 279, 445, 735, 1152 since the onset of the experiment. sSOAs, sSOA-surrounding sands, and control sands were then collected over a 3 year time series to investigate *in situ* biodegradation of SOAs and microbial community dynamics. Total enviromental DNA was extracted from the source material (sSOA at day 0), incubated SOAs, SOA-surrounding sands, and control sands and used for downstream analysis.

### Diversity and composition of microbial communities in SOAs

**Figure 2:**
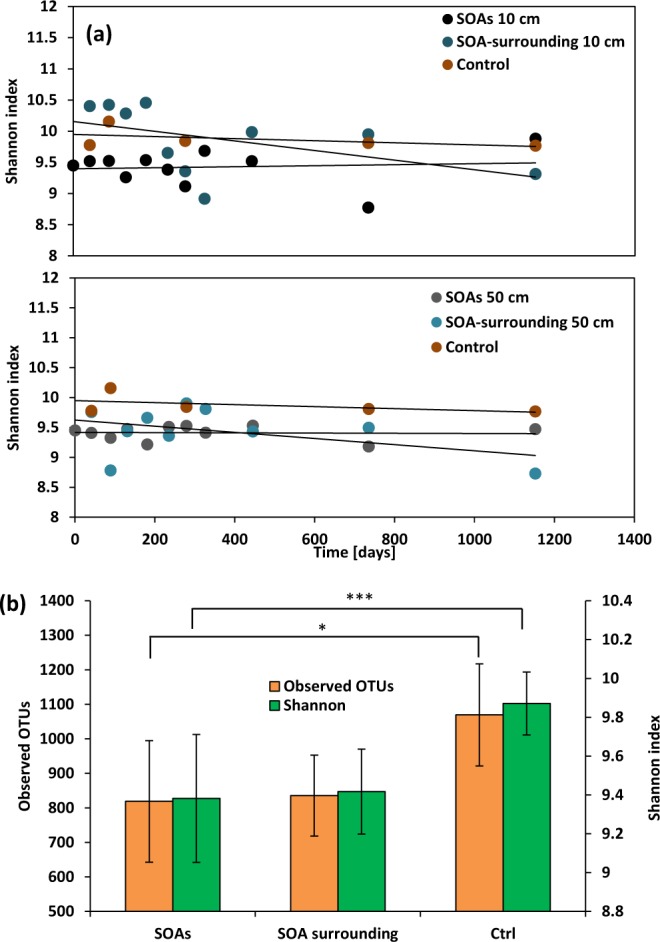
Diversity of microbial communities in sediment oil agglomerates (SOAs), SOA-surrounding sands, and control sands over the 3 year time course. Incubation time is represented as days after SOA deployment. (**a**) Alpha-diversity is calculated based on Shannon indices and (**b**) the number of observed OTUs (Student’s t test: p-value = 0.015* and 0.001***).

No significant trends in alpha diversity were observed over the time course of the experiment for microbial communities associated with SOAs and SOA-surrounding sands. Alpha diversity as determined by Shannon indices showed substantial variation (Fig. 2a) in SOAs and SOA-surrounding sands from both sediment depths, while diversity in control sands was more consistent with time. Whereas a previous study of buried small oil particles and oil films in the supratidal zone of Pensacola Beach (PB) showed a >50% reduction of Shannon indices during the initial 6 months after oil came ashore^17^, here we detected little to no reduction of Shannon indices in the SOAs. We attribute the lack of diversity change to the fact that the source material used in this study was already colonized by an established community of hydrocarbon-degrading bacteria. However, statistical analysis showed that Shannon indices in SOAs were distinct from those of control sands. The mean number of observed OTUs declined by 23% in SOAs and SOA-surrounding sands (827 ± 147 observed OTUs) in comparison to control sands (1069 ± 148 observed OTUs) (Fig. 2b).

**Figure 3:**
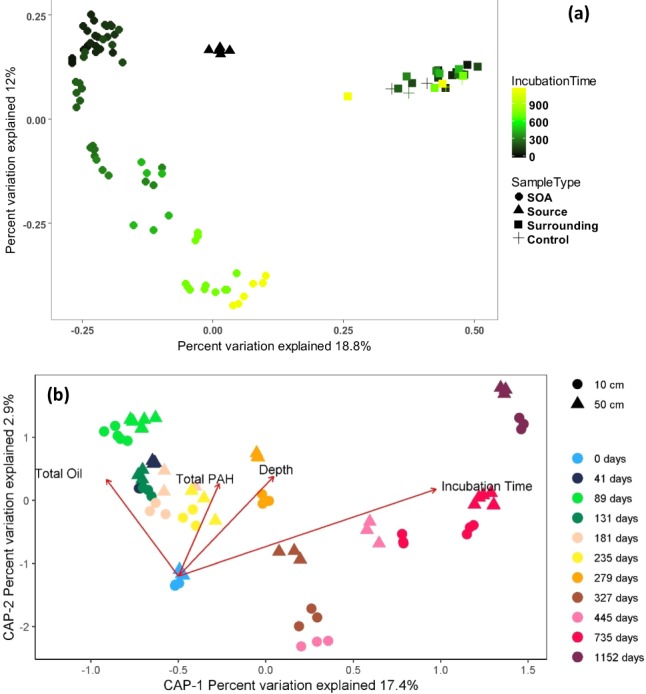
Effects of SOAs on Pensacola Beach microbial community. (**a**) The principal coordinates analysis PCoA and (**b**) constrained analysis of the principal coordinates (CAP) of bacterial communities. The ordination of the microbial community was constrained by the experimental variables to show how these factors affect the microbial community. The arrow’s length and direction indicate factors that have a significant effect on the microbial community organization.

In this study, the source material used for the experiment was produced from SOAs that had freshly formed from MC252-oil mousse washing onto the shore within a few months after the DWH oil spill (end of June 2010), and thus had a similar hydrocarbon composition as the weathered mousse^32^. SOAs formed as the mousse was stranded and mixed with beach sand. Microbial communities in the SOAs were distinct from those present in SOA-surrounding sands or in control sands, as determined by beta diversity of SSU rRNA genes according to the Bray Curtis distance metric (Fig. 3a). SOA community composition more closely resembled the source material in the beginning of the experiment and then strongly diverged from the source material and surrounding control sands across the time series, showing no evidence of recovery (Fig. 3a). To further examine the relationships between beta diversity and oil contamination, a canonical analysis of principal coordinates (CAP) analysis was performed exclusively on SOAs over time. For the first 6 months of the time series, community structure was strongly linked to total petroleum hydrocarbon concentrations, indicating that the input of labile hydrocarbon compounds was driving microbial community dynamics more than time or depth. Across intermediate time scales, after labile hydrocarbons were depleted, beta diversity appeared to vary with the concentration of polycyclic aromatic hydrocarbons. Subsequently, the influence of petroleum hydrocarbons diminished from the end of year 1 to year 3, and communities were structured by depth and time (Fig. 3b).

**Figure 4:**
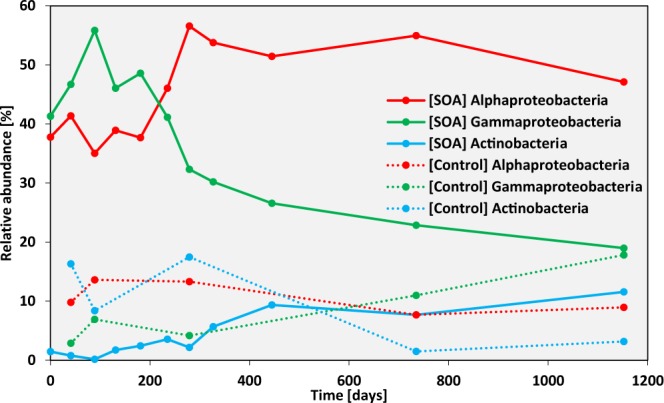
Relative abundance of *Alphaproteobacteria* (red), *Gammaproteobacteria* (green), and *Actinobacteria* (light blue) in SOAs (solid) and uncontaminated control sands (dotted) over the 3 year time course. Abundance is determined based on the total SSU rRNA gene sequences retrieved.

The impact of oil contamination on microbial community composition was further examined in buried SOAs by investigating the dynamics of specific taxa over the 3-year time series. The mean of relative abundances from the two different depths were pooled for further analysis. A clear succession of microbial populations was observed in SOAs with time, whereas these same taxa were much lower in relative abundance and showed no noticeable pattern in SOA-surrounding sands and control sands (Fig. 4). At the phylum and class level, microbial communities in SOAs were dominated by *Gammaproteobacteria* (42 to 58% relative abundance), *Alphaproteobacteria* (38 to 58%), and *Actinobacteria* (up to 10%) (Fig. 4). Few studies are available from microbial communities of oil emulsions or SOAs/ tar balls and taxonomic characterization in previous work was often limited to the phylum or class level^9,24,33^. In an investigation of 3 mousse samples collected off the coast of Louisiana, Liu and Liu (2013) observed a high relative abundance, up to 75%, of either *Alphaproteobacteria* or *Gammaproteobacteria*, similar to the SOAs studied here^33^. In previous work on Louisiana beaches impacted by the DWH spill, Urbano *et al*. (2013) observed enrichment of *Gammaproteobacteria* and *Alphaprotebacteria* in tar balls collected in the drier supratidal zone, while *Deltaproteobacteria* were detected in tar balls from the wetter intertidal zone^9^. Bacosa *et al*. (2016) observed an enrichment of hydrocarbon-degrading bacteria, mainly *Gammaproteobacteria*, in tar balls collected within 13 months of the Texas city “Y” spill, although a phylum to class level classification of microbial communities was not provided in their study^24^. SOA communities appear to more closely resemble those of mousse rather than pristine sands or sands containing more diffuse oiled particles^17,34,35^. In particular, SOAs and mousse foster much higher levels of *Alphaproteobacteria* and *Actinobacteria*.

Similar to previous work in environments impacted by the DWH spill, the *Gammaproteobacteria* showed a maximum relative abundance of 58% early in the time course at approximately 100 days, whereas the *Alphaproteobacteria* peaked at 58% later at ~ 300 days, followed by a bloom of up to 10% *Actinobacteria* after 400 days^34–36^ (Fig. 4). Both *Gammaproteobacteria* (*Alcanivorax, Marinobacter*) and *Alphaproteobacteria* (*Rhodobacteraceae*) were enriched in Pensacola Beach sands after the Deepwater Horizon oil spill and were identified as key players in oil degradation in previous studies that monitored microbial community shifts after weathered oil contamination over a relatively shorter time period^34,35^. As in these studies of more diffuse oil contamination on shorelines^34,37^, groups of known hydrocarbon-degrading bacteria within the *Gammaproteobacteria* (e.g. *Alcanivorax*) responded first to oil in SOAs followed by members of the *Alphaproteobacteria* at later stages when recalcitrant oil hydrocarbons predominated. Major differences between the SOA time series presented here and time series of more diffuse oil contamination in buried sands^17,33,34^ are that microbial succession occurred over much longer time scales (>3 years) and no recovery occurred in the SOAs. In previous work at Pensacola Beach^17,34,35^, PHCs returned to background levels one year after oil came ashore, and a typical beach sand microbial community had reestablished that showed little to no evidence of oil hydrocarbon degradation potential.

**Figure 5:**
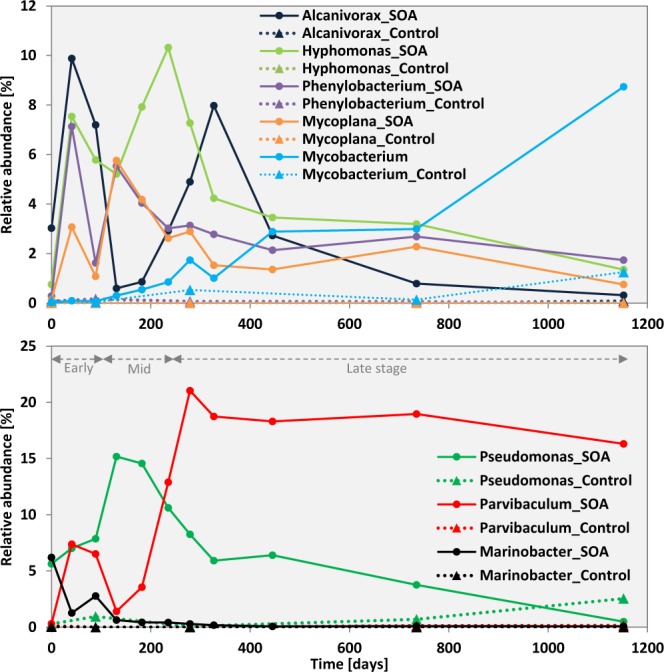
Relative abundance of taxa detected at the genus level in SOAs (solid) and uncontaminated control sands (dotted) over the 3-year time course. Abundance is determined based on the total SSU rRNA gene sequences retrieved.

At the order to genus level, three distinct maxima in microbial populations were observed with time in the SOAs, whereas these same taxa showed a much lower relative abundance in control samples (Fig. 5). Maxima in relative abundance are interpreted as microbial populations that respond to petroleum hydrocarbons available during the early (0–131 days of incubation), mid (131–235 days of incubation), and late stages (after 235 days of incubation) of the time series (Fig. 5; Supplemental Fig. 2). Early responders included the *Caulobacterales* within the class *Alphaproteobacteria* and *Oceanospirillales* within the class *Gammaproteobacteria* that increased in relative abundance during the first 100 days, from 0.5% to 12% and from 7% to 13%, respectively (Supplemental Fig. 2). At the genus level, the relative abundance of known alkane degraders such as *Alcanivorax*, *Hyphomonas*, *Phenylobacterium*, and *Mycoplana* increased early from 0.13–3% to 3–10% in SOAs and not in control sands (Fig. 5). *Alcanivorax* is known to degrade relatively short-chain alkanes and not being capable of degrading aromatic hydrocarbons^38,39^. In previous studies of oiled sands and tar balls, *Alcanivorax* spp. were the most abundant OTU in the oil-contaminated samples^24,34^. A high relative abundance of *Alcanivorax* (up to 20%) was also observed in a gravel beach after the Xingang oil spill in China^40^. The genus *Hyphomonas*, known to be able to utilize aromatic hydrocarbons, was enriched in oiled sand and microcosms with crude oil^35,41,42^. A DNA-based and stable isotope probing (SIP) study with [U-^13^C]anthraquinone from PAH-contaminated soil showed that the genus *Phenylobacterium* is responsible for anthraquinone degradation^43^. The genus *Mycoplana* is also known to degrade aromatic hydrocarbons^44,45^. Our observations are corroborated by petroleum hydrocarbon analysis, which revealed that short-chain alkanes (C_15_) and relatively low-molecular weight PAHs such as naphthalene were rapidly depleted over the first ~100 days when populations of early responders were enriched^32^.

Distinct microbial groups were enriched during the mid phase, between 131 and 235 days, of the time series. The genus *Pseudomonas* within the order *Pseudomonadales* was enriched from 5 to 15.2% after 131 days in SOAs, whereas *Pseudomonas* remained generally below 1% abundance in control sands. *Pseudomonas* has been reported to produce biosurfactant during PAH degradation and was also enriched in previous studies of oil-contaminated sands^34,35,46,47^. The relative abundance of *Rhizobiales*, *Actinomycetales*, and *Rhodospirillales* increased in the later stages of the time series after 235 days. *Parvibaculum* within the order *Rhizobiales* is known to degrade both aliphatic and aromatic hydrocarbons, and this genus was highly enriched in SOAs and not in control sands late in our time series, in agreement with previous work in oiled sands^35,48,49^. Relative abundance of *Mycobacterium*, a known PAH degrader, increased from <0.01% to 9% during the time series (Fig. 5). Evidence of *Mycobacterium* was also detected using a DNA fingerprinting approach from SOAs collected from supratidal zone of coastal headland beach in Louisiana^9^. *Mycobacterium* is the only genus shown to degrade 4-ring PAHs such as the chrysene observed in SOAs^9,50^. *Mycobacterium* is also known to be adapted to low moisture content and periods of desiccation^36^, which resembles the characteristics of dry SOAs^16^. Another known PAH-degrader *Stenotrophomonas* was a major microbial group associated with tar balls in the intertidal zone of a headland beach^9^, but its relative abundance in this study was very low at 0–0.22% throughout our time course. Members of the genus *Marinobacter*, a generalist group known to degrade both alkane and PAHs, were enriched in many oil-impacted environments^34,36,40,51,52^ but their abundances in SOAs decreased during the experiment from 6% to near 0% (Fig. 5). Petroleum hydrocarbon analysis showed that mid-chain alkanes (C_16_–C_30_), phenanthrene, and dibenzothiophene were degraded from ~100 days to ~300 days after the SOA burial. This implies that secondary responders e.g. *Pseudomonas* and some of early the responders such as *Hyphomonas* and *Mycoplana* were capable of degrading these hydrocarbon compounds. After approximately 300 days of incubation, primarily long-chain alkanes (C_30_–C_40_) remained to be degraded^32^, which coincided with the rapid increase of the relative abundance of *Parvibaculum*. A few isolates of the genus *Parvibaculum* such as *P. lavamentivorans* and *P. hydrocarboniclasticum* are known to be capable of utilizing *n*-alkanes or linear alkylbenzenesulfonates^49,53^, which implies degradation of long-chain alkanes by *Parvibaculum* at a later stage of incubation.

### The abundance of overall bacteria and diazotrophic communities in SOAs

**Figure 6:**
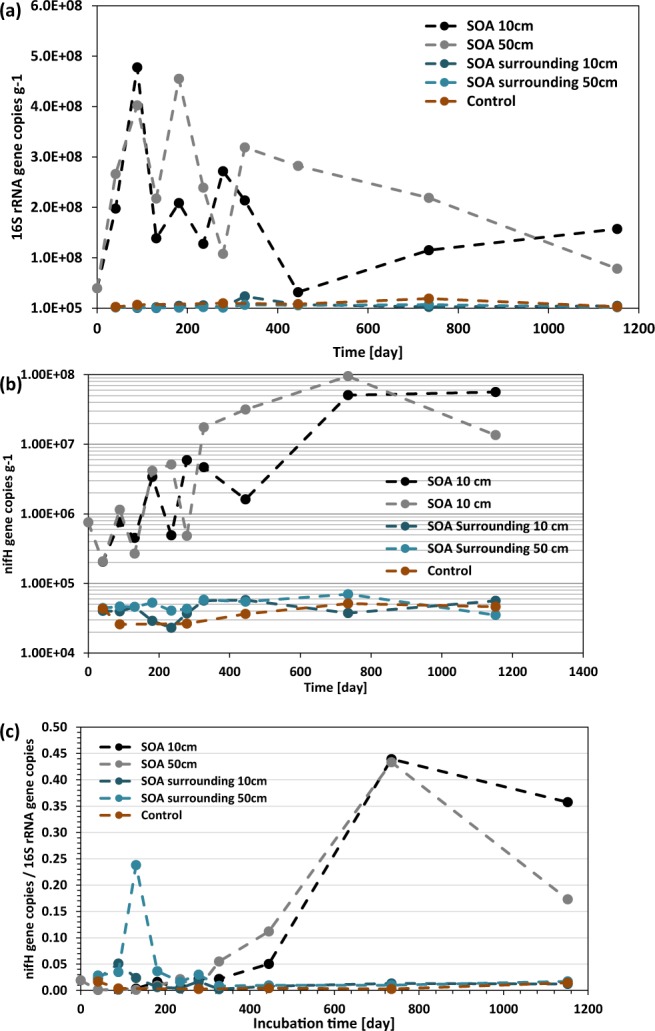
(**a**) SSU rRNA gene, (**b**) *nifH* gene abundance per gram sediment and (**c**) *nifH* gene abundance normalized to the abundance of SSU rRNA genes in 10 cm and 50 cm sediment depth intervals from SOAs and SOA-surrounding sands as well as control sands.

The abundance of SSU rRNA genes on average was three orders of magnitude higher in SOAs in comparison to the surrounding sands or control sands. This bacterial bloom indicates that SOAs are hotspots of microbial growth. In the SOAs, overall bacterial abundance increased from 3.4 × 10^7^ to 4.4 × 10^8^ copies g^−1^ during the first 89 days, whereas overall bacterial abundance in SOA-surrounding sands and control sands remained at 2.0 × 10^5^ to 2.4 × 10^7^ copies g^−1^. Characterization of the microbial communities associated with the SOAs by SSU rRNA gene sequencing, as discussed above, revealed a number of abundant groups that contain members known to be capable of nitrogen fixation (diazotrophy) including the *Rhizobiales*, *Frankiales*, *Rhodobacterales*, and *Rhodospirillales*, and we hypothesized that microbial nitrogen fixation would be enhanced with time. In order to estimate nitrogen fixation potential during *in situ* SOA incubation in Pensacola Beach sand, the abundance of genes encoding nitrogenase enzyme (*nifH*), the best studied molecular marker for nitrogen fixation^54^, was quantified. Results revealed that both SSU rRNA gene and *nifH* abundance in SOAs differed from those of SOA-surrounding sands and control sands (Fig. 6). Initially, the ratio of *nifH* to SSU rRNA gene abundance remained 0–0.02 for the first 300 days and then showed a large increase to 0.44 towards the latter stages of the time course, suggesting that diazotrophs bloomed after 1 year. The abundance of *nifH* genes in SOAs then was one to three orders of magnitude higher in comparison to the *nifH* abundance in the surrounding sands and control sands (7.6 × 10^5^ to 8.4 × 10^7^ copies g^−1^ in SOAs; 2.3 × 10^4^ to 7 × 10^4^ in surrounding; 2.6 × 10^4^ to 5.1 × 10^4^ in control). Previous studies have shown that SOAs contain extremely high C/N ratios indicative of nitrogen limitation^16^. In addition, the results from this SOA experiment are corroborated by previous studies of more diffuse oil in sands at Pensacola Beach, which also showed elevated nitrogenase abundance in oil-contaminated sand layers^35^. Results indicate that a paucity of nitrogen as evidenced by a high C/N ratio triggered microbial nitrogen fixation to produce bio-available nitrogen from atmospheric nitrogen.

**Figure 7:**
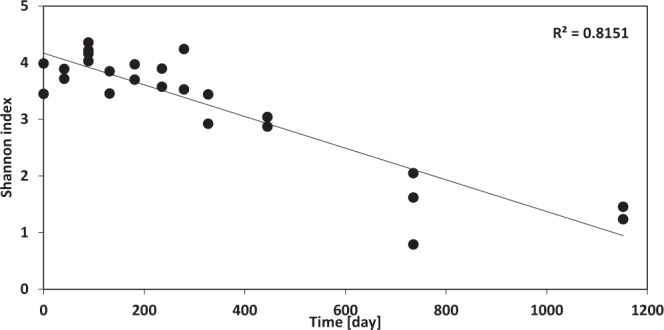
Alpha-diversity of nitrogenase gene sequences over the three-year time course of SOA samples incubated in Pensacola Beach sand.

Sequencing of *nifH* amplicons was conducted to further elucidate the microbial groups responsible for diazotrophy in the oiled dry beach environment. Diazotroph microbial diversity as determined by Shannon indices decreased over the time series (Fig. 7). At the phylum to class level, results showed that the *Alphaproteobacteria* and *Actinobacteria* were the most dominant diazotroph groups in SOAs (relative abundances of 64–71% and 15–21%, respectively) across the time series (Supplemental Fig. 3a). At the order level, members of the *Rhizobiales* (36–44% relative abundance) of the *Alphaproteobacteria* and the *Frankiales* of the *Actinobacteria* (15–21%) were the most abundant throughout time series. The late bloom of diazotroph abundance is concurrent with the maximum relative abundance of *Rhizobiales and Frankiales* in the times series, determined by sequencing of SSU rRNA as well as *nifH* amplicons, which peaked after 400 days. At the genus level, *Methylobacterium* within the order *Rhizobiales* was the most abundant diazotroph group, which constituted from 25 to 33% of *nifH* gene relative abundance (Supplemental Fig. 3b). *Methylobacterium* increased rapidly from 25% relative abundance after 445 days of incubation to 31% abundance at the end of the time course. The second most abundant diazotroph group was *Frankiales* which comprised up to 15–21% relative abundance.

Members of the *Rhizobiales* as well as *Frankiales* are well known as nitrogen-fixing symbionts associated with plant roots^55^, and free-living members of the *Rhizobales* are also thought to catalyze nitrogen fixation in a variety of ecosystems^56^. *Methylobacterium* was shown to grow on PAHs as well as produce biosurfactants in oil-contaminated systems^57,58^. Recently, it was shown that *Frankia* grows with PAHs as the sole carbon source and contains genes for alkane degradation^59^. *Methylobacterium* was also abundant in oil mousse collected from the sea surface and in salt marshes in the northern Gulf impacted by the DWH oil spill^33,60^. Therefore, multiple lines of evidence indicate a close coupling of petroleum hydrocarbon degradation to nitrogen fixation in SOAs. In our time series, nitrogen appears to become limiting after the first year, resulting in selection for microbial populations capable of coupling nitrogen fixation to hydrocarbon degradation.

### Inferred metagenomic analysis

Based on microbial community composition in SOAs as determined by SSU rRNA gene amplicon sequencing, inferred metagenomic analysis was performed to assess the metabolic potential of the communities across the time series. Given the abundance of hydrocarbon-degraders and diazotrophs in the time course, our analysis focused on functional genes for hydrocarbon degradation and nitrogen fixation (Supplemental Fig. 4). The predicted relative abundance of alkane-1-monooxygenase (*alkB*) genes peaked within the first approximately 90 days post burial and then decreased rapidly, suggesting that relatively simple hydrocarbon substrates such as alkanes were utilized by bacteria at this early stage of the time series. Microbial groups that were predicted to contribute to alkane degradation include members of the *Rhodobacteraceae*, *Pseudomonadaceae*, *Alcanivoraceae*, and *Alteromonadaceae*. The predicted relative abundance of naphthalene 1,2-dioxygenase genes (*nahAc*, *ndoB*, *nbzAc*, *dntAc*) and other PAH dioxygenase genes (*nidA*, *nidB*) reached a maximum later in the time series, at approximately 200 and 300 days post initiation, respectively. *Pseudomonas* was predicted to contribute to the degradation of more recalcitrant, aromatic hydrocarbons later in the time series. Lastly, predicted relative abundance of nitrogenase genes increased rapidly after 400 days and peaked at approximately 750 days, indicating enhanced bacterial nitrogen fixation at a later stage of SOA incubation. Microbial groups that are predicted to contribute to nitrogenase gene abundance include *Rhizobiales*, *Rhodobacterales*, and *Rhodospirillales* (Supplemental Fig. 5).

Predictions from PICRUSt were corroborated by the chemical evolution of petroleum hydrocarbons, as determined in our companion study^32^, along with temporal trends in the abundance of overall bacteria and diazotrophs. The predicted abundance of hydrocarbon degradation genes peaked during the first 400 days in parallel with overall bacterial abundance as well as the degradation of alkanes and aromatic compounds. Predictions of alkane monooxygenase (*alkB*) abundance showed good agreement with the consumption of short chain (C_15_) alkanes, with both showing maximum changes during the first 100 days. Between 100 and 400 days, the consumption of longer chain alkanes (C_18_-C_22_) was not concurrent with the predicted *alkB* abundance, indicating that other enzyme pathways are responsible for the degradation of these compounds. Finally, the predicted abundance of nitrogenase shows good agreement with the observed nitrogenase abundance, with both showing the largest increases between 400 and 750 days. Thus, multiple evidence support the coupling of hydrocarbon degradation to diazotrophy in SOAs.

## Conclusions

Macroscopic sediment-oil agglomerates (SOAs) were formed when MC252 oil from the Deepwater Horizon disaster reached the shores of the northern Gulf of Mexico and interacted with the sediment. Hydrocarbon-degrading bacteria were enriched and a succession of microbial populations was observed that paralleled the chemical evolution of the petroleum hydrocarbons over longer time scales (>3 years) in comparison to previous work on more diffuse oil contamination in beach sands. We provide evidence of bacterial blooms in SOAs, underlining that these large aggregates are hotspots of microbial growth. Our quantification of diazotrophs in large aggregates shows that nitrogen-fixing taxa predominate in oil-degrading microbial communities during the late stages of the time course when nutrients likely become depleted. The coupling of nitrogen fixation to hydrocarbon degradation thus represents a key process for the microbial decomposition of macroscopic oil aggregates.

## Materials and Methods

### Sample collection and experimental design

Sediment oil agglomerates (SOAs) were collected at Pensacola Beach, FL, USA (30.3261 N, 87.1744 W) on 30 June, 2010. SOAs were homogenized, and then filled into 3.8 cm diameter-stainless steel meshballs producing standardized SOAs. After determining the initial masses of each standardized SOA, 10 filled meshballs were attached in pairs to a PVC pipe (1.3 cm diameter) at 10 cm intervals (Fig. 1). Ten of these meshball arrays were buried in the supratidal zone at Pensacola Beach on 22 October, 2010 such that the meshballs were located at 10, 20, 30, 40 and 50 cm sediment depth (Fig. 1 and Supplemental Fig. 6). The arrays were retrieved 41, 89, 131, 181, 235, 279, 327, 445, 735, and 1152 days after burial, and the mass of each SOA was determined again before freezing at −20 °C in clean glass jars for further microbial community analysis. Together with the arrays, sandy sediments from the region surrounding each deployed SOA were also collected in order to identify possible impacts to indigenous microbial communities. This study analyzed SOAs and associated sand buried at 10 and 50 cm sediment depth. Control sand without oil contamination was collected from the surface of a nearby pristine sand dune at the same study site after 41, 89, 279, 445, 735, 1152 days of incubation.

### Nucleic acid extraction and microbial community analysis

Total genomic DNA was extracted from SOAs using a MoBio PowerSoil DNA isolation kit (MoBio Laboratories, Carlsbad, CA) with slight modifications from the manufacturer’s protocol. Briefly, 0.25 g of thawed SOA sample was placed into a 2 ml bead tube and homogenized for 1 min using a Talboys High Throughput Homogenizer (Troemner, Thorofare, NJ). Overall microbial communities and nitrogen-fixing prokaryotes were characterized by targeting SSU rRNA and nitrogenase (*nifH*) genes, respectively. PCR amplification of SSU rRNA genes was performed using 515 F (5′-GTGCCAGCMGCCGCGGTAA-3′) and 806 R (5′-GGACTACHVGGGTWTCTAAT-3′) primers as described by the Earth Microbiome Project (http://www.earthmicrobiome.org/emp- standard-protocols/dna-extraction-protocol/) for Illumina sequencing^61^. For *nifH*, the primer set IGK3 (5′-GCIWTHTAYGGIAARGGIGGIATHGGIAA-3′) and DVV (5′-ATIGCRAAICCICCRCAIACIACRTC-3′) was used for PCR amplification as described previously^62^. PCR products were barcoded using an Access Array Barcode Library (Fluidigm, South San Francisco, CA), purified using the E.Z.N.A Cycle Pure Kit (Omega Bio-tek, Norcross, GA), and pooled based on DNA concentration. Purified and pooled PCR amplicons were sequenced on an Illumina MiSeq platform at the DNA services facility at the University of Illinois at Chicago (https://rrc.uic.edu/). Sequence analysis was accomplished using the software QIIME ver. 1.9.1^63^ and Mothur ver. 1.38.0^64^. Sequences with quality score below 20 were removed using Mothur ver. 1.38.0 and clustered into operational taxonomic units (OTUs) by 97% and 92% sequence identity for SSU rRNA and *nifH* genes^62^, respectively using UCLUST^65^ implemented in QIIME ver. 1.9.1. Representative sequences were aligned against the SILVA ver. 123 database (https://www.arb-silva.de/) or *nifH* reference alignments database^62^, and chimeric sequences were removed using UCHIME^65^ implemented in Mothur ver. 1.38.0. These high-quality sequences were taxonomy assignment to the SILVA SSU rRNA or *nfH* reference alignments database with the RDP classification algorithm with a minimum confidence threshold of 50%. (https://www.arb-silva.de/, Gaby *et al*., 2018). The resultant OTU table was scaled using the CSS algorithm implemented in QIIME ver. 1.9.1^66^. The microbial diversity calculations and statistical analyses were performed with default R functions or with “phyloseq” and “vegan” R packages^67–69^. For alpha diversity analysis, Shannon indices were calculated with QIIME ver. 1.9.1. To assess shifts in the diversity and community composition over time, a Bray-Curtis distance matrix was calculated from the rarefied OTU table and used for a principal coordinate analysis (PCoA) and canonical analysis of principal coordinates (CAP) analyses. The oil effect on community similarity and dispersion was estimated with a PERMANOVA and BETADISP statistical tests with 1,000 permutations. Additionally, CAP analysis was performed to assess the correlation between microbial community structure and the following variables: Incubation.Time + Depth + Total.Oil + Total.PAH + Total.Alkens using “capscale” function in R package vegan. The significance of the CAP models was tested using the “permutest” function in a vegan package with 999 permutations. Finally, the Mantel correlation test with 10,000 permutations was applied to determine the similarity between the patterns of the chemical components and microbial communities.

Based on microbial community composition in SOAs as determined by SSU rRNA gene amplicon sequencing, Phylogenetic Investigation of Communities by Reconstruction of Unobserved States (PICRUSt) was employed in order to predict metagenome functional content of differentially abundant OTUs^70^ and predict Kyoto Encyclopedia of Genes and Genomes (KEGG) Ortholog functional profiles. The OTU table was normalized using the software QIIME ver. 1.9.1^63^, and each OTU was divided by SSU rRNA gene abundance. The resultant OTU table was used to create the final metagenome functional predictions. All raw sequences have been uploaded to NCBI under Bioproject PRJNA450618.

### Quantitative molecular analyses

To evaluate quantitative changes in the abundance of overall bacterial communities and nitrogen-fixing prokaryotes (or diazotrophs), quantitative PCR was performed with PowerUp SYBR Green Mastermix (Applied Biosystems, Foster City, CA) and 331 F (5′-TCCTACGGGAGGCAGCAGT-3′)/515 R (5′-ATTACCGCGGCTGCTGG-3′) primers targeting bacterial SSU rRNA genes or PolF (5′‐TGCGAYCCSAARGCBGACTC‐3′)/PolR (5′‐ATSGCCATCATYTCRCCGGA‐3′) primers targeting the *nifH* marker gene for nitrogen-fixing *Bacteria* and *Archaea* as previously described^71–73^. All reactions were performed in triplicate and analyzed using StepOne Software v. 2.3. Serially diluted pGEM-T Easy Vector plasmids (Promega, Madison, WI) containing either a full-length *E. coli* SSU rRNA gene or *nifH* gene were used to generate standard calibration curves for quantification of gene abundances. The efficiencies of the quantitative PCR assay ranged from 95.3 to 101%.

## Supplementary information

Supplementary Figures

## References

[1] Reddy CM (2012). Composition and fate of gas and oil released to the water column during the Deepwater Horizon oil spill. Proc. Natl. Acad. Sci. USA.

[2] Zukunft, P. F. Summary report for sub-sea and sub-surface oil and dispersant detection: sampling and monitoring. *Oper. Sci. Advis. Team* (2010).

[3] Hayworth JS, Clement TP, Valentine JF (2011). Deepwater Horizon oil spill impacts on Alabama beaches. Hydrol. Earth Syst. Sci..

[4] Wang P, Roberts TM (2013). Distribution of Surficial and Buried Oil Contaminants across Sandy Beaches along NW Florida and Alabama Coasts Following the Deepwater Horizon Oil Spill in 2010. J. Coast. Res..

[5] Barron M, Awkerman J, Raimondo S (2015). Oil Characterization and Distribution in Florida Estuary Sediments Following the Deepwater Horizon Spill. J. Mar. Sci. Eng..

[6] Michel J (2013). Extent and Degree of Shoreline Oiling: Deepwater Horizon Oil Spill, Gulf of Mexico, USA. PLoS One.

[7] Nixon Z (2016). Shoreline oiling from the Deepwater Horizon oil spill. Mar. Pollut. Bull..

[8] Fingas M, Fieldhouse B (2009). Studies on crude oil and petroleum product emulsions: Water resolution and rheology. Colloids Surfaces A Physicochem. Eng. Asp..

[9] Urbano M, Elango V, Pardue JH (2013). Biogeochemical characterization of MC252 oil: Sand aggregates on a coastal headland beach. Mar. Pollut. Bull..

[10] Lemelle KR, Elango V, Pardue JH (2014). Distribution, characterization, and exposure of MC252 oil in the supratidal beach environment. Environ. Toxicol. Chem..

[11] Aeppli C (2012). Oil weathering after the Deepwater Horizon disaster led to the formation of oxygenated residues. Environ. Sci. Technol..

[12] Gustitus SA, Clement TP (2018). Formation, Fate, and Impacts of Microscopic and Macroscopic Oil-Sediment Residues in Nearshore Marine Environments: A Critical Review. Rev. Geophys..

[13] Hayworth JS, Prabakhar Clement T, John GF, Yin F (2015). Fate of Deepwater Horizon oil in Alabama’s beach system: Understanding physical evolution processes based on observational data. Mar. Pollut. Bull..

[14] Yin F, John GF, Hayworth JS, Clement TP (2015). Long-term monitoring data to describe the fate of polycyclic aromatic hydrocarbons in Deepwater Horizon oil submerged off Alabama’s beaches. Sci. Total Environ..

[15] Operational Science Advisory Team (OSAT-2). Summary Report for fate and effects of remnant oil in the beach environment. *Gulf Coast Incid. Manag. Team* 1–36 (2011).

[16] Elango V, Urbano M, Lemelle KR, Pardue JH (2014). Biodegradation of MC252 oil in oil:sand aggregates in a coastal headland beach environment. Front. Microbiol..

[17] Huettel M (2018). Degradation of Deepwater Horizon oil buried in a Florida beach influenced by tidal pumping. Mar. Pollut. Bull..

[18] John GF, Han Y, Clement TP (2016). Weathering patterns of polycyclic aromatic hydrocarbons contained in submerged Deepwater Horizon oil spill residues when re-exposed to sunlight. Sci. Total Environ..

[19] Kiruri LW, Dellinger B, Lomnicki S (2013). Tar balls from deep water horizon oil spill: Environmentally persistent free radicals (EPFR) formation during crude weathering. Environ. Sci. Technol..

[20] Tao Z, Bullard S, Arias C (2011). High numbers of Vibrio vulnificus in tar balls collected from oiled areas of the north-central Gulf of Mexico following the 2010 BP Deepwater Horizon oil spill. Ecohealth.

[21] White HK (2016). Long-term weathering and continued oxidation of oil residues from the Deepwater Horizon spill. Mar. Pollut. Bull..

[22] Radović JR (2014). Assessment of photochemical processes in marine oil spill fingerprinting. Mar. Pollut. Bull..

[23] Prince RC (2003). The roles of photooxidation and biodegradation in long-term weathering of crude and heavy fuel oils. Spill Sci. Technol. Bull..

[24] Bacosa HP, Thyng KM, Plunkett S, Erdner DL, Liu Z (2016). The tarballs on Texas beaches following the 2014 Texas City “Y” Spill: Modeling, chemical, and microbiological studies. Mar. Pollut. Bull..

[25] Salleh AB, Ghazali FM, Abd Rahman RNZ, Basri M (2003). Bioremediation of Petroleum Hydrocarbon Pollution. Indian J. Biotechnol..

[26] Dibble JT, Bartha R (1979). Effect of Environmental Parameters on the Biodegradation of Oil Sludge. Appl. Environ. Microbiol..

[27] Coty VF (1967). Atmospheric nitrogen fixation by hydrocarbon-oxidizing bacteria. Biotechnol. Bioeng..

[28] Toccalino PL, Johnson RL, Boone DR (1993). Nitrogen limitation and nitrogen fixation during alkane biodegradation in a sandy soil. Appl. Environ. Microbiol..

[29] Chen YP, Lopez-de-Victoria G, Lovell CR (1993). Utilization of aromatic compounds as carbon and energy sources during growth and N2-fixation by free-living nitrogen fixing bacteria. Arch. Microbiol..

[30] Prantera MT, Drozdowicz A, Leite SG, Soares A (2002). Degradation of gasoline aromatic hydrocarbons by two N2 -fixing soil bacteria. Biotechnol. Lett..

[31] Medina-Bellver JI (2005). Evidence for *in situ* crude oil biodegradation after the Prestige oil spill. Environ. Microbiol..

[32] Bociu, I. *et al*. Decomposition of sediment-oil-agglomerates in a Gulf of Mexico sandy beach. *Sci Rep* **9**, 10071 10.1038/s41598-019-46301-w (2019). 10.1038/s41598-019-46301-w PMC6624294 31296898

[33] Liu Z, Liu J (2013). Evaluating bacterial community structures in oil collected from the sea surface and sediment in the northern Gulf of Mexico after the Deepwater Horizon oil spill. MicrobiologyOpen.

[34] Kostka, J. E. *et al*. Hydrocarbon-degrading bacteria and the bacterial community response in Gulf of Mexico beach sands impacted by the deepwater horizon oil spill. *Appl. Environ. Microbiol.* **77**, 7962–7974 10.1128/AEM.05402-11 (2011). 10.1128/AEM.05402-11 PMC3208977 21948834

[35] Rodriguez-R, L. *et al*. Microbial community successional patterns in beach sands impacted by the Deepwater Horizon oil spill. *ISME J* **9**, 1928–1940 10.1038/ismej.2015.5 (2015). 10.1038/ismej.2015.5 PMC4542042 25689026

[36] Lamendella R (2014). Assessment of the deepwater horizon oil spill impact on gulf coast microbial communities. Front. Microbiol..

[37] Alonso-Gutierrez J (2009). Bacterial communities from shoreline environments (Costa da Morte, northwestern Spain) affected by the Prestige oil spill. Appl. Environ. Microbiol..

[38] Liu C, Shao Z (2005). Alcanivorax dieselolei sp. nov., a novel alkane-degrading bacterium isolated from sea water and deep-sea sediment. Int. J. Syst. Evol. Microbiol..

[39] Schneiker S (2006). Genome sequence of the ubiquitous hydrocarbon-degrading marine bacterium Alcanivorax borkumensis. Nat. Biotechnol..

[40] Zhang K (2017). Periodically spilled-oil input as a trigger to stimulate the development of hydrocarbon-degrading consortia in a beach ecosystem. Sci. Rep..

[41] Coulon F, McKew BA, Osborn AM, McGenity TJ, Timmis KN (2007). Effects of temperature and biostimulation on oil-degrading microbial communities in temperate estuarine waters. Environ. Microbiol..

[42] Kappell AD (2014). The polycyclic aromatic hydrocarbon degradation potential of Gulf of Mexico native coastal microbial communities after the Deepwater Horizon oil spill. Front. Microbiol..

[43] Rodgers-Vieira EA, Zhang Z, Adrion AC, Gold A, Aitken MD (2015). Identification of anthraquinone-degrading bacteria in soil contaminated with polycyclic aromatic hydrocarbons. Appl. Environ. Microbiol..

[44] Brinda Lakshmi M, Muthukumar K, Velan M (2012). Immobilization of Mycoplana sp. MVMB2 Isolated from Petroleum Contaminated Soil onto Papaya Stem (Carica papaya L.) and Its Application on Degradation of Phenanthrene. Clean - Soil, Air, Water.

[45] Brinda Lakshmi M, Anandaraj VP, Velan M (2013). Bioremediation of Phenanthrene by Mycoplana sp. MVMB2 Isolated from Contaminated Soil. Clean - Soil, Air, Water.

[46] Deziel E (1996). Biosurfactant production by a soil pseudomonas strain growing on polycyclic aromatic hydrocarbons. These include: Biosurfactant Production by a Soil Pseudomonas Strain Growing on Polycyclic Aromatic Hydrocarbons. Appl. Environ. Microbiol..

[47] Prabhu Y, Phale PS (2003). Biodegradation of phenanthrene by Pseudomonas sp. strain PP2: novel metabolic pathway, role of biosurfactant and cell surface hydrophobicity in hydrocarbon assimilation. Appl. Microbiol. Biotechnol..

[48] Lai Q (2011). Parvibaculum indicum sp. nov., isolated from deep-sea water. Int. J. Syst. Evol. Microbiol..

[49] Rosario-Passapera R (2012). Parvibaculum hydrocarboniclasticum sp. nov., a mesophilic, alkane-oxidizing alphaproteobacterium isolated from a deep-sea hydrothermal vent on the East Pacific Rise. Int. J. Syst. Evol. Microbiol..

[50] Khan AA, Kim SJ, Paine DD, Cerniglia CE (2002). Classification of a polycyclic aromatic hydrocarbon-metabolizing bacterium, Mycobacterium sp. strain PYR-1, as Mycobacterium vanbaalenii sp. nov. Int. J. Syst. Evol. Microbiol..

[51] Atlas RM (2015). Oil Biodegradation and Oil-Degrading Microbial Populations in Marsh Sediments Impacted by Oil from the Deepwater Horizon Well Blowout. Environ. Sci. Technol..

[52] Wang J (2016). Biodegradation of dispersed Macondo crude oil by indigenous Gulf of Mexico microbial communities. Sci. Total Environ..

[53] Schleheck D, Tindall BJ, Rosselló-Mora R, Cook AM (2004). Parvibaculum lavamentivorans gen. nov., sp. nov., a novel heterotroph that initiates catabolism of linear alkylbenzenesulfonate. Int. J. Syst. Evol. Microbiol..

[54] Gaby, J. C. & Buckley, D. H. A comprehensive evaluation of PCR primers to amplify the nifH gene of nitrogenase. *PLoS One* **7** (2012). 10.1371/journal.pone.0042149 PMC3405036 22848735

[55] Erlacher A (2015). Rhizobiales as functional and endosymbiontic members in the lichen symbiosis of Lobaria pulmonaria L. Front. Microbiol..

[56] Sellstedt A, Richau KH (2013). Aspects of nitrogen-fixing actinobacteria, in particular free-living and symbiotic frankia. FEMS Microbiol. Lett..

[57] Salam LB, Obayori OS, Raji SA (2015). Biodegradation of Used Engine Oil by a Methylotrophic Bacterium, Methylobacterium Mesophilicum Isolated from Tropical Hydrocarbon- contaminated Soil Biodegradation of Used Engine Oil by a Methylotrophic Bacterium, Methylobacterium Mesophilicum Isolated. Pet. Sci. Technol..

[58] Nzila A, Thukair A, Sankara S, Chanbasha B, Musa MM (2016). Isolation and characterization of naphthalene biodegrading Methylobacterium radiotolerans bacterium from the eastern coastline of the Kingdom of Saudi Arabia. Arch. Environ. Prot..

[59] Rehan, M. & Swanson, E. Frankia as a Biodegrading Agent Frankia as a Biodegrading Agent, 10.5772/61825 (2016).

[60] Engel, A. S. *et al*. Salt marsh bacterial communities before and after the Deepwater Horizon oil spill. *Appl. Environ. Microbiol*. **83** (2017). 10.1128/AEM.00784-17 PMC5626990 28778895

[61] Caporaso JG (2012). Ultra-high-throughput microbial community analysis on the Illumina HiSeq and MiSeq platforms. ISME J..

[62] Gaby, J. C. *et al*. Diazotroph community characterization via a high-throughput nifH amplicon sequencing and analysis pipeline. *Appl Environ Microbiol* **84**, e01512-17. 10.1128/AEM.01512-17 (2018). 10.1128/AEM.01512-17 PMC5795091 29180374

[63] Caporaso JG (2010). QIIME allows analysis of high-throughput community sequencing data. Nat. Methods.

[64] Schloss PD (2009). Introducing mothur: Open-source, platform-independent, community-supported software for describing and comparing microbial communities. Appl. Environ. Microbiol..

[65] Edgar RC, Haas BJ, Clemente JC, Quince C, Knight R (2011). UCHIME improves sensitivity and speed of chimera detection. Bioinformatics.

[66] Paulson JN, Stine OC, Bravo HC, Pop M (2013). Differential abundance analysis for microbial marker-gene surveys. Nat. Methods.

[67] R Core Team, R Foundation for Statistical Computing, Vienna, A. R: A language and environment for statistical computing. Available online at, https://www.r-project.org/ (2018).

[68] Dixon P (2003). Computer program review VEGAN, a package of R functions for community ecology. J. Veg. Sci..

[69] Mcmurdie PJ, Holmes S (2013). phyloseq: An R Package for Reproducible Interactive Analysis and Graphics of Microbiome Census Data. PLoS One.

[70] Langille MGI (2013). Predictive functional profiling of microbial communities using 16S rRNA marker gene sequences. Nat. Biotechnol..

[71] Warren, M. J. *et al*. Molybdenum-based diazotrophy in a Sphagnum peatland in northern Minnesota. *Appl Environ Microbiol* **83**, e01174-17. 10.1128/AEM.01174-17 (2017). 10.1128/AEM.01174-17 PMC5561275 28667112

[72] Carrell AA (2019). Experimental warming alters the community composition, diversity, and N 2 fixation activity of peat moss (Sphagnum fallax) microbiomes. Glob. Chang. Biol..

[73] Kolton M, Marks A, Wilson RM, Chanton JP, Kostka JE (2019). Impact of Warming on Greenhouse Gas Production and Microbial Diversity in Anoxic Peat From a Sphagnum -Dominated Bog (Grand Rapids, Minnesota, United States). Front. Microbiol..

