# Supplementary material for: Succession of microbial populations and nitrogen-fixation associated with the biodegradation of sediment-oil-agglomerates buried in a Florida sandy beach
Source: Sci Rep. 2019 Dec 18;9:19401. doi: 10.1038/s41598-019-55625-6 (PMC6920467; doi:10.1038/s41598-019-55625-6)
Supplement: Supplementary file 1 — Supplementary Figures [file 41598_2019_55625_MOESM1_ESM.docx]

**Succession of microbial populations and nitrogen-fixation associated with the biodegradation of sediment-oil-agglomerates buried in a Florida sandy beach**

Boryoung Shin^1^, Ioana Bociu^2^, Max Kolton^3^, Markus Huettel^2^, and Joel E. Kostka^1,3*^

^1^*School of Earth and Atmospheric Sciences, Georgia Institute of Technology, Atlanta, GA, USA*

*^2^Department of Earth, Ocean and Atmospheric Science, Florida State University, Tallahassee, FL, USA*

*^3^School of Biology, Georgia Institute of Technology, Atlanta, GA, USA*

*Corresponding author: Joel E. Kostka, joel.kostka@biology.gatech.edu

Supplemental Figures

Supplemental Figure 1. Relative abundance change of classes of Alphaproteobacteria, Gammaproteobacteria, Acidobacteria, and Actinobacteria from (a) SOA-surrounding sands and (b) control sands.

Supplemental Figure 2. Relative abundance of taxa detected at the order level in sediment oil agglomerates (SOAs) over the 3 year time course. Abundance is determined based on the total SSU rRNA gene sequences retrieved.

Supplemental Figure 3. (a) Microbial community structure at the class level based on nifH gene sequences retrieved from SOAs over the three year time course. (b) Relative abundance of the genera Methylobacterium, Frankia, and Novosphingobium based on nifH gene sequence analysis over the three year time course.

Supplemental Figure 4. Predicted relative abundance of functional genes over the three year time course as determined by PICRUST. Relative abundance of genes encoding (a) alkane-1-monooxygenase (AlkB) – K00496, (b) naphthalene 1,2-dioxygenase subunit alpha – K14579, PAH dioxygenase large subunit – K11943, PAH dioxygenase small subunit – K11944, (c) nitrogenase iron protein NifH – K02588, nitrogen fixation protein NifB – K02585, nitrogenase molybdenum-iron protein NifN – K02592, and nitrogenase delta subunit – K00531.

Supplemental Figure 5. Predicted community composition of diazotrophs determined by inferred metagenomic analysis (PICRUST) of nifH genes.

Supplemental Figure 6. SOA in situ incubation in Pensacola Beach. Ten SOA-filled meshballs were attached with their chain to a PVC pipe that kept them at a defined location and defined sediment depth from 5 cm to 55 cm. Duplicate SOA-filled meshballs were attached at each sediment depth at 10 cm depth intervals. SOA arrays were buried at Pensacola Beach on October 22, 2010 in the high dry beach that could only be reached by seawater during big storm events.
